# Supplementary material for: Are carbon emissions trading and green financial instruments synergistic? -Comprehensive quantitative research based on content analysis
Source: PLoS One. 2024 Mar 7;19(3):e0298601. doi: 10.1371/journal.pone.0298601 (PMC10919601; doi:10.1371/journal.pone.0298601)
Supplement: S1 Data — (ZIP) [file pone.0298601.s001.zip › Data/地级市Z_F工作报告.zip!/║╙─╧/Σ≡║╙╩╨/└┤╫╘╣1⁄2╓┌║┼í╛┬φ┐╦╩2╛▌═°í┐_2017.docx]

各位代表：

现在，我代表市人民政府，向大会报告工作，请予审议，并请各位政协委员和其他列席人士提出意见。

一、2016年及本届政府工作回顾二号首长

去年，面对复杂严峻的外部环境和艰巨繁重的发展任务，在市委的坚强领导下，在市人大、市政协的监督支持下，全市上下认真学习习近平总书记系列重要讲话精神，全面贯彻党中央、国务院大政方针，深入落实省委、省政府工作部署，主动适应经济发展新常态，以新发展理念为引领，坚持稳中求进工作总基调，深入推进供给侧结构性改革，着力发挥优势打好“四张牌”，持续深化六项重大攻坚，“十三五”经济社会发展实现了良好开局。

——生产总值、固定资产投资、金融机构存款余额三项重要指标突破千亿元大关。全市生产总值、固定资产投资、金融机构存款余额分别达到1077.9亿元、1060.4亿元和1043.2亿元，漯河经济社会发展站上了新的起点，我们的家底更加厚实。

——多数经济指标增速保持或进入全省第一方阵。规模以上工业增加值、固定资产投资、社会消费品零售总额、居民人均可支配收入分别增长9%、16.7%、12.4%和8.1%，均居全省第5位；一般公共预算收入增长11%，居全省第4位；金融机构存、贷款余额分别增长23%和27.1%，居全省第1位；我市在全省经济社会发展目标考核评价综合排序中名列第8位，比上年前移2位。

——环保攻坚取得突破性进展。主要污染物浓度全面下降，空气优良天数达到190天，比上年增加40天，比全省平均水平多增加26天，7条省控河流断面水质全部实现稳定达标。

——脱贫攻坚年度目标圆满完成。贫困人口识别精准率和群众满意率均在95%以上，全年有2.6万贫困人口实现稳定脱贫、54个村退出贫困村，我市在全省脱贫工作成效考核中居第2位。

尤为振奋的是，我市在国务院开展的贯彻中央重大政策措施第三次大督查中再次受到表扬，成为全省唯一受到表扬、全国唯一连续两年受到表扬的城市，漯河在全国的影响力持续提高。

过去的一年，我们主要做了以下工作：

（一）稳增长、保态势，发展的稳定性进一步增强。加强经济运行过程管理，建立市政府常务会议政治学习制度，扎实开展“两学一做”学习教育。自觉接受各方监督，省人大代表建议、政协提案及748件市人大代表议案、建议和政协提案全部办结；加强行政监察和审计监督，对11个单位开展了审计；组织16个单位主要负责人接受网友在线访谈，扎实开展涉企科室评议等活动。深入推进法治政府建设，依法办理市政府行政复议案件46件；全面推进政务公开，公开各类政府信息1.7万余条。认真落实中央八项规定精神和省委、市委贯彻意见，扎实开展“四项突出问题”专项治理等活动，政风行风持续好转。

各位代表！2016年目标任务的顺利完成，标志着本届政府各项工作圆满收官。过去的五年，面对种种困难挑战和群众热切期盼，全市上下在抢抓机遇中乘势而上，在爬坡过坎中克难前行，在攻坚转型中铸就辉煌，经济社会发展取得了令人鼓舞的重大成就！

——这五年，综合实力大幅提升。2016年，全市生产总值、固定资产投资、社会消费品零售总额、金融机构存款余额、金融机构贷款余额分别是2012年的1.3倍、2倍、1.7倍、1.8倍和2.1倍；规模以上工业企业实现利润310.4亿元，是2012年的1.4倍；一般公共预算收入完成76亿元，是2012年的1.8倍。

——这五年，六项重大攻坚实现突破。项目招大引强取得重大成效，共引进投资超10亿元的重大项目66个，完成投资190亿元。工业优化升级取得新进展，高新技术产业增加值达到172.5亿元，是2012年的3.2倍。骨干企业群体快速壮大，规模以上工业企业达到729家，比2012年增加177家，双汇母公司万洲国际成为全国唯一一家进入世界500强的食品企业。服务业发展加快，第三产业增加值占生产总值的比重达到27.4%，比2012年提高4.8个百分点；服务业税收达到55.5亿元，是2012年的2.6倍。县域经济实力增强，临颍、舞阳两县的经济总量、一般公共预算收入分别是2012年的1.3倍和2.3倍。城市建设力度持续加大，市区建成区面积达到80平方公里、常住人口达到76万人，分别比2012年增加18平方公里和14万人。

——这五年，发展动力持续增强。新培育国家级高新技术企业40家、省级创新型企业9家，新建省级企业技术中心和工程技术研究中心32个；科技进步对经济增长的贡献率达到56%，比2012年提高6个百分点。取消、下放479项行政审批事项；市场主体达到9.2万户，比2012年增加3.2万户。累计实际利用外资38.7亿美元、利用省外资金940亿元，外贸进出口总值30.5亿美元，分别是上个五年的2.7倍、2.6倍和2.1倍 。

——这五年，城乡面貌显著变化。市区新建、改造134条道路、7座沙澧河大桥和一批供气、供水、排水、环卫等设施，污水日处理能力达到40万吨，垃圾无害化处理率超过98%。城市旧城区、棚户区和城中村改造成效明显，累计建设安置房317万平方米。新建国家4A级景区3个。城市绿化覆盖率、绿地率分别达到40.2%和34%，人均公共绿地面积达到11.8平方米。新开通城市公交线路9条，新配置新型节能环保车辆260台，群众乘坐公交更加方便。成功创建国家卫生城市，全国文明城市中考取得较好成绩。新建改造农村公路1525公里，新解决95万农村人口的饮水安全问题，成功创建6个国家级生态示范村、17个省级生态示范乡镇、257个省级农村人居环境示范村。

——这五年，人民生活明显改善。居民人均可支配收入达到18887元，比2012年增加6186元。城市低保标准每月515元，农村低保标准每月247元，分别比2012年提高215元和117元；农村五保集中供养标准每年4000元、分散供养标准每年3000元，分别比2012年提高1520元和1500元；新建保障性住房8.8万套，人均住房面积达到46平方米，比2012年提高3.1平方米；新增学生学位3.2万个、医院病床6641张、养老床位2226张。

这些成绩极大地提高了漯河在全省发展大局中的地位和作用，极大地增强了全市人民的自豪感和自信心，全面建成小康社会在沙澧大地展现出更加美好的前景。成绩来之不易，这是以习近平同志为核心的党中央正确领导的结果，是省委、省政府和市委科学运作的结果，是市人大、市政协监督支持的结果，是全市人民团结奋斗的结果。在此，我代表市人民政府，向工作在各条战线的工人、农民、知识分子、干部，向驻漯部队、武警消防官兵、公安民警以及社会各界人士，致以崇高的敬意！向所有关心支持漯河经济社会发展的同志们、朋友们，表示衷心的感谢！

在看到成绩的同时，我们也清醒地认识到，前进道路上还面临不少困难和问题，政府工作与人民群众的期待还有不小差距。一是经济稳定增长的基础还不够牢。产业结构不合理，骨干企业不多，经济整体竞争力不强；财政增收乏力，收支矛盾加大；实体经济困难较多，融资难、融资贵问题比较突出，经济金融风险逐步暴露，一些地方政府债务隐患持续增加。二是发展动能转换还不够快。部分领域改革急需深化，“放管服”改革的协同性、联动性不够，有些改革举措和政策落实不到位，开放水平急需提升，企业创新能力急需提高，大项目支撑作用急需增强，动能转换的“四梁八柱”要真正搭建起来尚需时日，供给侧结构性改革任务还很艰巨。三是城乡建设的效果还不够好。城乡基础设施欠账较多，公共服务水平较低；中心城市带动能力不强，城乡一体化发展推进不快，农村人居环境有待继续改善，农村仍是全面建成小康社会的突出“短板”。四是生态环境改善的程度还不够大。环境质量基础依然脆弱，大气、水和土壤污染防治形势仍很严峻，环保攻坚任重道远。五是人民群众的幸福感还不够强。经济总量还不大，人均生产总值还低于全省平均水平；城乡居民收入不高，就业门路不多，人民群众关心的住房、教育、文化、医疗、养老等方面的问题没有得到很好解决，扶贫攻坚任务还很重，食品药品安全、安全生产、涉法信访、征地拆迁等方面还存在不少薄弱环节。六是政府治理现代化水平还不够高。政府职能转变还不彻底，科学民主依法决策还不到位；一些政府部门工作作风不扎实，服务质量和效率不高，行政执法中不规范、不公正、不文明现象依然存在，部分工作人员存在着懒政怠政、为官不为等问题，涉企服务、群众办事难等问题仍较突出；一些领域腐败问题时有发生。对此，我们一定高度重视，敢于担当，采取有力有效措施，竭尽全力加以解决，决不辜负全市人民的重托和期望！

二、今后五年总体要求和奋斗目标侯卫东官场笔记

今后五年，我市面临着继续加快发展、做大经济总量，推进提质发展、加速经济转型，促进共享发展、增加人民福祉等多重任务，必须全面贯彻党的十八大和十八届三中、四中、五中、六中全会精神，深入落实习近平总书记系列重要讲话和调研指导河南工作时的重要讲话精神，统筹推进“五位一体”总体布局和协调推进“四个全面”战略布局，按照市第七次党代会总体部署，坚持发展是第一要务，以新发展理念为引领，以提高发展质量和效益为中心，以推进供给侧结构性改革为主线，以全面深化六项重大攻坚为统揽，着力壮大总量增强实力，着力优化结构加快转型，着力改善民生促进和谐，持续推进一区两城一中心建设，确保再上“两个大台阶”，四个方面“走在全省前列”，如期全面建成小康社会，为中原更加出彩作出更大贡献。

今后五年的奋斗目标是：

——综合经济实力再上一个大台阶。经济保持中高速增长，生产总值年均增长8%，主要经济指标增速高于全省平均水平，人均生产总值达到或接近全省平均水平，经济效益、生态效益和社会效益全面提升，我市在全省发展大局中的地位和作用更加凸显。

——人民生活水平再上一个大台阶。居民收入增长与经济增长同步，城镇新增就业15万人、农村劳动力转移就业新增10万人，贫困村全部退出、贫困人口稳定脱贫，上学难、看病贵问题得到更好解决，全市人民的生活更加幸福、日子越过越红火。

——产业转型升级走在全省前列。先进制造业和现代服务业成为产业主体，高新技术产业产值占规模以上工业产值的比重达到42%，产业高端化、绿色化、智能化、融合化发展水平明显提高，农业现代化成为全省排头兵，产业市场竞争力显著增强。

——发展动能转换走在全省前列。创新能力明显增强，科技进步对经济增长的贡献率达到60%，研发经费投入强度超过2%。改革全面深化，成为全省体制改革和机制创新的先行区。开放平台和开放机制基本完善，中原地区内陆开放高地基本形成。

——城乡建管水平走在全省前列。常住人口城镇化率达到56%以上，60%的行政村成为美丽宜居村庄，成功创建全国文明城市，积极创建国家健康城市、生态园林城市，城市管理、农村人居环境改善成为全省标兵，城乡一体化发展成为全省标杆。

——生态环境建设走在全省前列。生产生活绿色、低碳水平显著提高，污染治理取得显著成效，能源消费总量和强度控制在省定目标以内，城市和农村废弃物资源化水平大幅提升，中原水城、生态绿城基本建成，漯河成为全省最宜居宜业的城市之一。

实现上述目标，必须把握好几个方面：一是必须深入贯彻党中央治国理政新理念新思想新战略。把创新、协调、绿色、开放、共享的发展理念贯彻到政府各项工作中，把认识、适应、引领新常态贯穿到经济社会发展的各领域，把提高供给质量作为推进供给侧结构性改革的主攻方向，增强发展动力，厚植发展优势，筑牢发展根基。二是必须坚持一张蓝图绘到底。在省委、省政府正确领导下，在历届市委、市政府打下的良好工作基础上，一任接着一任干，不动摇、不懈怠，不跑偏、不折腾，不提不切实际的空口号，多做补短板、打基础、利长远的实在事，努力创造经得起检验的实绩。三是必须全面深化六项重大攻坚。围绕创新驱动全面深化，推动发展动力加快转换；围绕增创优势全面深化，提高综合竞争力；围绕补齐短板全面深化，提高发展的协调性和平衡性；围绕打造亮点全面深化，提高漯河影响力。四是必须撸起袖子加油干。压实各方责任，层层传导压力，勇于奋发作为，全力干事创业，以实干推动发展，以实干赢得未来。

各位代表！目标催人奋进，漯河前程似锦。只要我们不忘初心，恪尽职守，砥砺奋进，一步一个深深的脚窝，扑下身子抓落实，宏伟蓝图就一定能够实现，漯河的明天一定会更加美好！

三、2017年主要目标和重点工作市委班子

做好今年的政府工作，必须把握“总遵循”，深入贯彻习近平总书记系列重要讲话和调研指导河南工作时的重要讲话精神，全面落实党中央治国理政新理念新思想新战略，打好“四张牌”；必须坚持“总基调”，突出稳增长保态势，在稳的前提下更加注重提质发展，加快优化结构转型升级；必须用好“总抓手”，全面深化六项重大攻坚，保持经济平稳健康发展和社会和谐稳定。

今年的主要目标是：生产总值增长8%以上，一般公共预算收入增长7.5%，固定资产投资增长13%，社会消费品零售总额增长12%，进出口总值保持稳定，城镇新增就业3万人以上，居民人均可支配收入增长8%，居民消费价格涨幅3%左右，质量效益指标稳步提升，节能减排、环境保护等约束性指标完成省定任务。

实现今年的目标，必须坚持问题导向，注重长短结合，突出重点，综合施策，精准发力，协同推进，统筹抓好五项工作。

（一）坚持以稳定经济增长和促进转型提质发展为目标，持续优化供给和扩大需求。优化供给结构，释放需求潜力，强力推动产业转型发展攻坚，以推进食品行业一二三产业融合发展为突破口，带动全市产业高端化、绿色化、智能化、融合化发展。

继续抓好“去降补”。着力去产能，再清理一批“僵尸企业”，严控过剩行业新上产能。着力去库存，扩大公积金受益覆盖面，提高棚改货币化安置比例，健全购租并举的住房制度，落实好解决市中心城区规划管理历史遗留问题的政策措施，促进房地产市场平稳健康发展。着力去杠杆，推动企业加快兼并重组、债务重组，盘活存量资产，优化负债结构。着力降成本，深入落实国务院降成本方案和省里50条措施，着重降低生产要素和制度、交易成本；扩大小微企业享受减半征收所得税优惠范围，削减行政事业性收费，降低各类中介评估费用，推动产业集聚区企业享受大企业直供电优惠政策。着力补短板，加快提升公共服务、基础设施、创新发展、资源环境、开放条件、体制机制等支撑能力。

扩大有效需求。多方筹措资金，强化土地供应，加快重点项目建设，确保165个市定重点项目完成投资500亿元以上；认真落实鼓励支持民间投资的政策措施，进一步调动民间投资的积极性。加快发展医养结合、健康护理、体育健康、绿色消费和乡村休闲、全域旅游等消费业态，建设一批文化旅游精品项目和健康养老产业园区，积极推进龙江生态城文化大观园项目建设；引导企业增品种、提品质、创品牌，更好满足消费升级需求。在重点推动食品、农产品加工企业深度开拓国际市场的同时，加快培育新的出口增长点，积极扩大服务贸易。

优化工业结构。以绿色安全、知名品牌为引领，推动食品产业融合发展，重点依托双汇集团等龙头企业和经济技术开发区、临颍休闲食品基地等平台，实施一批产业升级项目；以发挥特色优势、实现局部突破为重点，推动装备制造业集群化发展，重点支持远大住建、鸿路集团、双建管桩、亿通新能源汽车等龙头企业项目建设，抓紧引进一批配套企业，加快建设绿色装配式建筑产业基地和新能源汽车生产基地；以延链补链、兼并重组为途径，推动传统产业转型发展，重点抓好盐化工精深加工、钛化工业和造纸业兼并重组；以提高研发能力、加快成果转化为核心，推动医药医疗产业快速发展，重点支持生物医药、医疗器械和市场需求较大的康复器械产业发展壮大；以龙头带动、集群配套为抓手，推动电子信息产业倍增发展，重点在有可能在我市形成完整产业链的细分产业，有针对性地招引龙头企业；以创新引领、重点突破为方向，推动新材料、新能源产业加速发展，重点支持协鑫集团多晶硅、中钢研蓝宝石等项目加快建设。深化“工业1351工程”，启动实施“十百千”亿级企业和产业集群培育工程，用三至五年时间，培育一批年主营业务收入超10亿元、100亿元、1000亿元的骨干企业和产业集群。启动实施“小升规”工程，争取50家以上小微企业成为规模以上企业。实施“双百”技改工程，抓好100家重点企业100个技改项目。试点引入市场机制，创新产业集聚区运营模式。深入实施提质转型创新发展三年行动计划，争创全省“十强”“十快”“十先”产业集聚区。

发展壮大服务业。充分发挥区位优势，优化物流产业空间布局，加快推进临港产业园现代物流核心区重大项目建设。补齐金融保险业短板，基本完成农信社改制组建农商行，支持双汇财务公司等地方金融机构发展，争取浦发银行、兴业银行、中信银行入驻我市，推动一批企业在新三板、四板挂牌。深入挖掘电子商务发展潜力，争创国家级电子商务示范基地。提升发展商贸等传统服务业，加快发展文化产业、旅游休闲、信息服务、商务会展、健康养老、楼宇经济、总部经济等现代服务业。推动生产性服务业向商务中心区集聚、生活性服务业向特色商业区集聚，带动服务业“两区”提质扩容、争星进位。

深化农业供给侧结构性改革。深入实施藏粮于地、藏粮于技战略，完善农业基础设施，新建高标准粮田12万亩以上，稳步提高粮食综合生产能力。加快农业科技创新和重大技术推广，推进良种育繁推一体化。着力调优种养结构、调强加工能力、调大经营规模、调长产业链条，调减玉米种植面积，大力发展优质专用小麦、优质油料、优质草畜、优质林果和小辣椒、食用菌等特色经济作物，加快发展美丽生态畜牧业，推进绿色安全优质高品质肉基地建设，积极创建全国畜牧业绿色发展示范市。大力发展现代农业产业化联盟，构建以龙头企业为核心、农业合作社为纽带、专业大户和家庭农场为基础的新型农业经营组织联盟，促进产业对接、要素联接、利益链接，实现各类新型农业经营主体互促共赢。加快发展农村集体经济。深入推进农村改革，开展房地一体的农村权籍调查，颁发统一的不动产权证书，落实承包土地所有权、承包权、经营权分置政策，深化集体林权、供销社等改革，健全农村“双创”促进机制，培育更多新型职业农民。

（二）坚持以人的城镇化为核心，持续促进城乡区域协调发展。牢固树立全市一盘棋的理念，统筹中心城区、县城、小城镇和新农村建设，着力构建城乡一体、区域联动的发展新格局。

加快农业转移人口市民化。深化户籍制度改革，重点推动一批已经在城市就业或生活的常住人口、一批城中村城郊村和产业集聚区内的人口、一批大中专院校在校学生、一批有意愿在城区落户的农民进城落户。全面推行居住证制度，保证居住证持有人在居住地享有与其他市民一样的基本公共服务。切实维护进城落户农民土地承包权、宅基地使用权、集体收益分配权。今年，常住人口城镇化率提高1.6个百分点，全市城镇化率突破50%。

加强城乡建设管理。从今年起，用三年时间，实施“三大行动计划”。实施功能区功能完善行动计划，按照市城乡一体化示范区、经济技术开发区、西城区三个功能区和一批产业集聚区、商务中心区、特色商业区的发展定位，加快完善功能，彰显区域特色；实施集中供暖行动计划，分步推进城区集中供暖工程；实施旧城区和城中村、城郊村改造行动计划，启动6个城中村改造工程，提升完善建成区内的多层住宅小区。实施六大重点工程。实施水系建设工程，完成生态水系规划编制，加快推进沙澧河大二期开发工程和西城区水系建设，开工建设幸福渠水系、城乡一体化示范区水系工程，提升经济技术开发区中水利用景观项目；实施交通畅通工程，开工建设市区至临颍、舞阳的快速通道，实施107国道西移新建工程、东三环路和北三环路建设工程，新建、改造、打通一批城市道路，建成太白山路沙河桥和澧河桥、中山路上跨立交桥；实施生态绿化工程，编制生态绿城建设规划和市区周边镇绿地系统规划，建设主通道防护林和一批街头游园；实施环保设施建设工程，加快建设经济技术开发区污水处理厂二期、马沟污水处理厂等一批污水处理设施，争取开工建设市区垃圾焚烧发电厂、餐厨垃圾处理厂和建筑垃圾消纳处理场；实施城市功能综合提升工程，持续推进五角场、荣昌商业广场、西城区商业核心区、长江国际广场、龙江生态城、五里庙城市综合体等节点街区建设，加快智慧城市建设；实施城市便民工程，对市区基础设施进行整体改造提升，重点抓好市区道路改造提升、背街小巷改造、交通标识标牌提升、公厕建设安装、城区居民安全饮水、路灯改造提升、智能立体停车楼建设、公交场站建设和市场改造，统筹考虑城市整体规划设计，推动城市节点、环线道路、基础设施、立面改造、景观绿化等与周围的环境相适应、色彩相协调、整体相融合。紧抓全省实施百城建设提质工程的机遇，着力增强临颍、舞阳两县的承载功能、吸纳功能、服务功能、生态功能和文化功能。从今年起，分期、分批对10个市区周边镇实施镇容镇貌综合整治，组团式配置公共设施和交通基础设施，建成宜居宜业新镇区，实现与中心城区融合发展；对两县和城区的其他镇区，突出现代农业和生态保护等功能，着力建设生态型和服务型小城镇；选择一批具有特色资源、区位优势的区域，加快培育成为特色小镇。分类推进新农村建设，持续加强农村基础设施和基本公共服务体系建设，切实抓好农村人居环境改善，认真解决新型农村社区建设遗留问题。设立市、县两级城市管理和综合执法局，深入推进城市精细化管理，全力冲刺创建全国文明城市，让群众生活得更方便、更舒心。

推动区域协调发展。鼓励和支持各行政区、功能区在全市发展大格局中，立足优势、主动作为、打造特色、竞相发展。临颍县重点打造千亿工业新城、文明生态新城、豫中南有影响力的市场物流和电子商务基地。舞阳县重点加快建设中部地区盐化工产业基地、国家级现代农业示范基地、区域性商贸物流基地。郾城区重点打造全省有影响的健康产业、商贸流通、生态宜居、现代农业四个示范区和新能源汽车产业基地。源汇区重点打造全省知名的医疗器械产业基地、新兴的电子信息产业基地、品位时尚的豫中南城市休闲中心、独具魅力的特色商业区。召陵区重点打造工业经济强区、电子商务新区、文化旅游名区、新业态商业区、环境优美的生态宜居区、城乡发展和谐区。市城乡一体化示范区重点加快建设多式联运内陆港和临港产业园、文化教育园、都市生态农业园、豫中南对外开放和商贸物流中心，努力成为全省城乡一体化发展样板区。经济技术开发区重点加快建设百强食品工业基地、中小企业孵化创新基地、液压系统配套基地，综合实力尽快进入全省国家级开发区前列。西城区重点打造商贸金融中心、文化行政服务中心、低碳生态智慧新区、海绵城市新区，尽快建成代表漯河发展水平和城市形象的现代化新城区。

（三）坚持以生态建设和环境保护为重点，持续推进绿色发展。积极回应人民群众对良好生态环境的热切期盼，着力解决人民群众反映强烈的突出环境问题，推动生态环境质量持续好转。

抓好生态绿城建设。抓好生态修复、城市修补试点工作，实施林业生态市提升工程。打造森林生态系统，重点沿中心城区向外放射的8条道路两侧，建设20至100米的绿化带，形成生态绿色网络。打造湿地生态系统，重点建设沙河国家湿地公园、龙江湿地公园、三河湾湿地公园和舞阳县澧河河道湿地公园等项目。打造流域生态系统，重点建设沙河、澧河、颍河、黑河、汾河等生态涵养林带，加大凤凰岛综合开发力度，做好澧河饮用水水源地取水口上移等项目的前期工作。打造农田生态系统，高标准建设农田防护林体系，扎实推进土壤污染防治。打造城市生态系统，加快城市生态廊道和城市游园建设，扎实推进龙江路生态绿道等项目，绿化市区20条道路。

打好环境保护攻坚战。持续深化大气污染治理，确保全市PM10浓度均值控制在每立方米104微克以下，PM2.5浓度均值控制在每立方米67微克以下，空气优良天数达到201天以上。强力推进水污染治理攻坚，以市区建成区消除黑臭水体、保障饮水安全为重点，系统推进水污染防治、水生态保护和水资源管理，确保城市饮用水源地澧河取水水质达标率保持100%，7条省控河流断面水质综合达标率达到省定目标要求，实现天更蓝、水更净。

发展好循环经济。强化节能降耗，积极推进重点行业清洁生产和农村清洁能源建设，加快静脉产业园前期工作，实施重点领域效能提升计划，推进低碳示范点建设，发展节能环保产业。

（四）坚持以创新开放改革为途径，持续增强发展动力和活力。把培育新动能作为事关漯河前途命运的大事来抓，争当全省改革开放排头兵、创新发展先行者，展现“内陆特区”新辉煌。

大力实施创新驱动。壮大创新主体，实施创新龙头企业培育工程和“科技小巨人”企业培育工程，新培育国家级高新技术企业5家以上。搭建创新载体，支持企业研发中心建设，新建省级以上研发中心6家以上，新建省级公共检验检测中心3家，建好中科天剑食品研究院，支持职业技术学院、食品学院建设食品产业公共研发平台，组建双汇食品研究院，加快建设经济技术开发区全省区域产业创新基地。完善创新机制，改革科研人员分配机制，实行“科技券”制度，深入推进产学研合作，支持骨干企业牵头建立科技创新联盟。加大创新人才培养、引进力度。积极争取国家和省级科技创新项目，谋划实施一批市级科技创新项目。大力支持“双创”，推动各类创新主体创办新企业、开发新产品、改进新工艺，因地制宜建设一批众创空间、科技企业孵化器等创新平台，争创中国驰名商标和河南省著名商标10件以上。继续抓好商业模式、业态等创新，促进形成全方位、全过程的创新。厚植创新文化，形成鼓励创新、支持创造、宽容失败的良好氛围。

积极推进“双向”开放。主动融入“一带一路”战略和中国（河南）自贸区建设，推动好产品销出去、好项目引进来、有条件的企业走出去；加强与郑州航空港经济综合实验区等开放平台的精准对接，谋划建设郑州国际陆港豫中南集散中心；复制推广上海等自贸区改革试点经验。加快完善开放平台，推进进口肉类指定口岸清真肉类查验点建设，完善肉类口岸功能，对接河南电子口岸建设；申建漯河海关办事处和进境木材监管区；加快建设出口质量安全示范区；发展跨境电商，支持企业利用电商平台开拓国际市场；加大签约项目专项督查力度，进一步提高合同履约率、资金到位率和项目开工率。探索建立市场化专业化招商机制，依据我市重点发展的产业链及其分布的重点地区，谋划成立若干专业性招商组织和区域性招商机构，更有针对性地招商引资，承接企业的整体性转移和全产业链转移，再引进一批龙头型、基地型、出口型、中心性项目及配套项目。

全面深化各项改革。加快“放管服”改革，进一步完善行政许可标准化建设，再取消、下放55项行政审批事项，并对市本级保留的行政审批事项实行清单管理；创新监管方式，全面推行“双随机、一公开”制度和企业失信惩戒制度；推进公共资源交易平台整合；完善各级政府政务办事大厅服务功能，推动实体大厅与网上大厅融合；开展“减证便民”专项行动，实行“多证合一、一照一码”，最大限度方便群众。深化国有企业改革，将国企改革从工业企业转向全部国有企业，大力推进企业产权结构、组织结构、治理结构改革。深化投融资体制改革，加快政府投融资平台市场化转型，做大做强各级政府投融资平台；积极稳妥推广PPP模式，继续推进财政性资金基金化改革，用好用活各类基金，积极发展供应链金融。深化财政体制和国税、地税征管体制改革。深入落实支持非公有制经济发展的各项政策措施，着力构建“亲”“清”政商关系，更好激发非公有制经济发展活力。

（五）坚持以人民为中心的发展思想，持续增加人民群众福祉。把保障和改善民生作为政府工作的出发点和落脚点，在经济发展的基础上，多搞一些改善生产生活条件的项目，多办一些利民惠民的实事，多解决一些群众牵肠挂肚的难题，努力在学有所教、劳有所得、病有所医、老有所养、住有所居上取得新进展。

全面发展社会事业。优先发展教育，改善农村办学条件，提高办学质量和水平；持续抓好义务教育均衡发展，扩大城区优质教育资源；打响职业教育品牌，支持漯河医专、漯河食品职业学院申办本科院校，开工建设漯河职业技术学院产教园区、市第一中专新校区等项目，积极推进漯河技师学院新校区、漯河医专“两馆一场”等项目。加强与省内外知名高校合作，引进优质教育资源。加快卫生事业改革发展，抓好医疗、医保、医药、医院改革，全面推进城市公立医院综合改革试点，基本建立分级诊疗体系，深化县级公立医院综合改革，推行医保支付方式改革；实施智慧医疗工程，尽快形成覆盖全市基层医疗机构的医联体；加快市中心医院全科医师培训基地等项目建设，扎实推进基层医疗卫生机构标准化建设，扶持高等院校和医疗机构建成一批在豫中南地区领先的重点学科、重点科室，强力推进区域性医疗服务中心建设。大力发展文化体育事业，持续扩大许慎文化、贾湖文化、舞阳农民画和“幸福漯河”系列群众文化活动的影响，继续实施基层综合性文化服务中心建设提升工程，开工建设许慎文化学院、市科技馆、市地震监测台站，实施市气象科普馆提升工程，推动全民健身运动，继续承办一批大型体育赛事。积极推进市民之家、市美术馆等项目建设，加快中华字（辞）书博物馆等项目前期工作，建成投用城市展示馆、青少年综合实践基地，市文化馆、博物馆、图书馆等公益文化场馆向市民开放；建成市福利中心；新建一批社区老年人日间照料中心和农村幸福院；启动市殡仪馆搬迁；规划建设市档案馆新馆；抓好广播电视、新闻出版、史志等工作；加强社会主义核心价值观教育，大力弘扬“崇文尚德、务本图强”的漯河精神，深化行业道德实践和群众性精神文明创建，提高全社会文明程度。

强化就业再就业和社会保障工作。推动创业带动就业，推进全民技能振兴工程，重点支持大学生和返乡农民工创业，促进“零就业”家庭成员实现就业。全面治理拖欠农民工工资问题。实施全民参保登记计划，调整企业和机关事业单位退休人员养老金，城乡居民基本养老保险基础养老金标准提高到每人每月82元；完善城乡居民大病保险制度，落实困难群众大病补充医疗保险制度，实施统一的城乡居民基本医保制度，财政补助标准提高到每人每年450元；城乡低保财政月人均补差水平分别提高到不低于250元和142元；落实特困人员供养、残疾人扶助等制度，完善社会救助、社会养老服务体系，加强农村留守儿童、妇女、老人关爱服务；抓好全国适度普惠型儿童福利制度建设试点。

加强和创新社会治理。健全立体化社会治安防控体系，严格落实安全生产责任制，完善信访工作机制，提高公共应急管理水平，高度重视防范和化解金融风险、地方政府债务风险，最大限度地维护群众利益、公共利益和社会稳定。强化食品药品安全监管，实现食品安全监管全覆盖、无死角，打造全省食品安全一流城市。促进民族团结、宗教和睦，支持工会、共青团、妇联等人民团体发挥桥梁纽带作用。加强国防教育和国防后备力量建设，支持驻漯解放军、武警部队、预备役部队、民兵和人民防空建设，深入开展双拥共建，巩固军政军民团结，推动军民深度融合发展。

扎实办好“十大实事”。围绕社会关注的热点难点，再解决一批人民群众最关心、最直接、最现实的利益问题。一是深入推进精准扶贫、精准脱贫。脱贫攻坚重在精准，贵在精准，成败之举在于精准，要拿出“绣花”功夫抓扶贫，确保每针每线都下苦功，确保脱贫得到群众认可、经得起历史检验。市级财政扶贫投入达到中央和省级扶贫投入的40%以上，对有劳动能力的贫困人口实现就业培训全覆盖，将符合条件的对象全部纳入社会保障和补助范围，全年实现1.52万人稳定脱贫，舞阳县脱贫“摘帽”。新建农村道路300公里，实现贫困村道路畅通。二是市区和两县城市公交票价调整为1元。重污染天气红色预警期间，群众免费乘坐城市公交；市区新投放环保公交车辆200台，新建公交场站3座，优化公交线路3条，新开市区绕城环线2条，为群众出行提供更多便利。三是改造提升市区和县城建成区背街小巷；启动人民公园整体改造，建成开放临颍县黄龙湿地公园，扎实推进双汇广场精品园林工程，加快中原乡土植物文化博览园、经济技术开发区中心公园建设；新建改建公厕25座、垃圾中转站16座,进一步改善群众生活环境。四是加强学校信息化建设，基本实现中小学校互联网全覆盖、网络教学环境全覆盖；新建改扩建中小学25所，全市新增中小学学位7000个，进一步缓解中小学大班额问题。五是对市区29个老旧街区的供水管网进行改造，新铺设管网75公里，解决城市居民安全饮水问题。六是对市区主要路段交通标示牌进行更新，新增交通护栏35公里，施划交通标线15万平方米，升级改造老旧信号灯20处以上，在市区主要路口设置交通拥堵提示牌，治理市区易堵点10个，进一步缓解市区交通拥堵问题。七是关爱特殊群体。开展环卫工“爱心早餐”活动，为市区和县城2982名早班环卫工人免费提供早餐，对市县4820名环卫工人进行一次免费体检；对110名残疾儿童进行抢救性康复，对600名残疾人进行就业创业培训；培育100个“巧媳妇工程”示范点，进一步解决留守贫困妇女就业创业问题。八是有序推进全民健康工程。建成全市急性心肌梗死救治网络；开展65岁以上老人和6岁以下儿童免费注射流感疫苗、肺炎疫苗试点工作；建设完善村（社区）居民健身广场100个。九是实施棚户区改造项目16个，筹集安置房8335套；改造农村危房1850户；对市区老旧小区实行简易物业服务；启动居民小区充电桩和快递服务平台建设，持续提升居民小区公共服务水平。十是推进市区集中供暖行动计划，沙北恒瑞热电厂6月底前建成，10月底前完成解放路、龙江路12公里管网铺设和供热调试。

各位代表！改善民生没有终点，只有连续不断的新起点。尽管财力很紧、困难很多、任务很重，但我们仍要以更大的投入、更强的举措、更实的工作，持续加大保障和改善民生力度，尽最大努力让人民群众享受更多成果、得到更大实惠、过上更好生活！

四、全面提升政府工作水平

我们的政府是党领导下的人民政府，必须始终加强自身建设，做到为人民服务、对人民负责、受人民监督、让人民满意。

坚定政治方向。增强“四个意识”，坚决维护以习近平同志为核心的党中央权威，自觉在思想上政治上行动上同党中央保持高度一致，凡是党中央提倡的坚决响应，凡是党中央决定的坚决执行，凡是党中央禁止的坚决不做。推进“两学一做”学习教育常态化制度化，在真抓实做上深化拓展，用党章党规规范行为，用习近平总书记系列重要讲话精神武装头脑、指导实践、推动工作，确保中央大政方针、省委省政府和市委决策部署落地生根。

践行为民宗旨。始终把人民的期盼放在首位，把更多财力、更多资源投到民生改善上，把更多时间、更大精力投到为人民服务上。大力推进“互联网+政务服务”，让信息、数据多跑路，让企业、群众少跑腿。推动市政府行政服务中心与市直各部门及县区政府政务信息互联互通，加快形成全市统一的政务服务平台。完善企业服务长效机制，扎实开展“送政策、送温暖”活动，在市长热线开设企业服务专线，对企业实施精准帮扶。深入开展项目建设环境集中整治年活动，营造更加优良的发展环境。

严格依法行政。加快法治政府建设，严格依法履职，做到科学决策、民主决策、依法决策。自觉接受市人大法律监督、工作监督和市政协民主监督，认真办理人大代表议案、建议和政协提案，广泛听取各民主党派、工商联、无党派人士和人民团体的意见建议，加强行政监察和审计监督，主动接受司法监督、社会监督和舆论监督，加快推进“阳光漯河”建设。深入推进服务型行政执法工作，抓好“七五”普法教育，做好法律援助工作。深入推进政务公开，让政府更好地服务人民，让人民更好地监督政府。

改进工作作风。深入落实中央八项规定精神和省委、市委贯彻意见，一以贯之纠正“四风”。深入开展调查研究，多走乡间小路、多进企业大门、多听百姓呼声。进一步精简会议、文件，狠治庸政懒政怠政。强化担当尽责，面对矛盾不回避，面对困难不退缩，主动作为，勇挑重担；健全正向激励机制，为敢于担当者担当，为敢于负责者负责。坚持求实求效，对定下来的事、干起来的事，紧抓不放、一抓到底，善始善终、善作善成。

保持廉洁本色。认真落实全面从严治党要求，持续加强政府系统党风廉政建设和反腐败工作。加强对政府投资的重大项目、公共资金、公共资源、国有资产监管，依法依规依程序推进政府采购、项目招投标、土地招拍挂等活动。优化财政支出结构，严格财经纪律，压缩非重点支出，市本级“三公”经费降低10%，用政府的“紧日子”换取群众和企业的“好日子”。加强政府系统工作人员的教育管理，做到干净干事、清白做人。坚持反腐无禁区、全覆盖、零容忍，严肃查处侵害群众利益的不正之风。

各位代表！历史的画卷，总是在砥砺前行中展开；时代的鼓点，总是在克难奋进中擂响。站在新起点、取得新成绩、完成新任务、实现新目标，我们担负着神圣职责、身负着组织重托、肩负着人民信任，必须把发展的眼光放得更远，把奋斗的脚步踩得更实，拿出舍我其谁的气概、驰而不息的韧劲、功成不必在我的胸襟，用担当点亮希望之光，靠实干实现心中梦想！

各位代表！新征程充满机遇和挑战，新使命光荣而艰巨。让我们更加紧密地团结在以习近平同志为核心的党中央周围，在省委、省政府和市委的坚强领导下，拿出百倍的热情和干劲，全面深化六项重大攻坚，持续推进一区两城一中心建设，奋力谱写决胜全面小康的华丽篇章，以优异成绩迎接党的十九大胜利召开！
